# Parametric bootstrapping for biological sequence motifs

**DOI:** 10.1186/s12859-016-1246-8

**Published:** 2016-10-06

**Authors:** Patrick K. O’Neill, Ivan Erill

**Affiliations:** Department of Biological Sciences, University of Maryland, Baltimore County, 1000 Hilltop Circle, Baltimore, 21250 US

**Keywords:** DNA, Transcriptional regulation, Sequence motifs, Maximum entropy, Information content, Sampling methods

## Abstract

**Background:**

Biological sequence motifs drive the specific interactions of proteins and nucleic acids. Accordingly, the effective computational discovery and analysis of such motifs is a central theme in bioinformatics. Many practical questions about the properties of motifs can be recast as random sampling problems. In this light, the task is to determine for a given motif whether a certain feature of interest is statistically unusual among relevantly similar alternatives. Despite the generality of this framework, its use has been frustrated by the difficulties of defining an appropriate reference class of motifs for comparison and of sampling from it effectively.

**Results:**

We define two distributions over the space of all motifs of given dimension. The first is the *maximum entropy* distribution subject to mean information content, and the second is the *truncated uniform* distribution over all motifs having information content within a given interval. We derive exact sampling algorithms for each. As a proof of concept, we employ these sampling methods to analyze a broad collection of prokaryotic and eukaryotic transcription factor binding site motifs. In addition to positional information content, we consider the *informational Gini coefficient* of the motif, a measure of the degree to which information is evenly distributed throughout a motif’s positions. We find that both prokaryotic and eukaryotic motifs tend to exhibit higher informational Gini coefficients (IGC) than would be expected by chance under either reference distribution. As a second application, we apply maximum entropy sampling to the motif *p*-value problem and use it to give elementary derivations of two new estimators.

**Conclusions:**

Despite the historical centrality of biological sequence motif analysis, this study constitutes to our knowledge the first use of principled null hypotheses for sequence motifs given information content. Through their use, we are able to characterize for the first time differerences in global motif statistics between biological motifs and their null distributions. In particular, we observe that biological sequence motifs show an unusual distribution of IGC, presumably due to biochemical constraints on the mechanisms of direct read-out.

**Electronic supplementary material:**

The online version of this article (doi:10.1186/s12859-016-1246-8) contains supplementary material, which is available to authorized users.

## Background

The computational analysis of DNA, RNA and protein sequences is a cornerstone of bioinformatics, enabling the study of genomes and protein families and providing the scaffold for a broad range of algorithms used in the analysis of biological data [1]. At the molecular level, many biological processes rely on the recognition of specific sequence patterns, or motifs, that define specific interactions between biological molecules [2]. The ubiquity of these motifs has led to the proliferation of a vast array of bioinformatics algorithms dedicated to the discovery and study of these sequence elements and their evolution [2–8]. Transcription factors modulate gene expression by binding to DNA in the promoter region of regulated genes. This binding relies on the specific recognition of short (5–30 bp) DNA sequence motifs by the transcription factor (TF) and, therefore, the discovery and characterization of TF-binding motifs is essential to our understanding of transcriptional gene regulation [5, 9, 10].

The discovery of TF-binding motifs is based on the elucidation of statistically overrepresented sequence elements within a set of sequences known or suspected of harboring TF-binding sites (e.g. promoter regions of co-transcribed genes). Many algorithms for motif discovery have been developed over the years, but they can be broadly divided into word-based and probabilistic approaches [3]. Word-based methods rely on the enumeration of oligonucleotides [11], whereas probabilistic and machine learning approaches use models of varying complexity to represent TF-binding motifs, estimating model parameters through sampling or optimization techniques [2, 12–15]. Central to these approaches is the definition of a robust statistical framework, a TF-binding motif model and its enhancement with heuristics based on knowledge of the underlying biochemistry. Most motif discovery methods, for instance, can enforce symmetry in TF-binding motifs to enhance performance when the TF is known to bind as a homodimer [16]. Similarly, the canonical position-specific weight matrix (PSWM) model for TF-binding motifs, which assumes positional independence in the motif, can be extended to accommodate variable spacing or positional dependencies [17–19]. Determining the proper model for TF-binding motifs also plays a pivotal role in other aspects of their analysis, such as the search for TF-binding sites or the use of simulations to analyze TF-binding motif evolution [7, 8, 20–22].

In principle, many properties observed in experimentally determined collections of TF-binding sites could be used to enhance algorithms involved in the discovery, search and evolutionary simulation of TF-binding motifs through the inclusion of heuristics or the adoption of expanded models. A principled introduction of such enhancements, however, requires that the properties of naturally occurring TF-binding motifs be contrasted with those of random ensembles of motifs matching some of their defining statistics. Indeed, the practice of comparing empirical data to the statistics of random ensembles is common in other fields such as complex network analysis [23] and systems biology [24]. Comparatively little attention, though, has been paid to the problem of defining such ensembles for biological sequence motifs and designing algorithms to sample them efficiently.

As a measure of the optimal mean message length required to encode samples from a probability distribution, information content (IC) serves as a unifying statistic of sequence conservation [25, 26]. Here we propose and characterize two different algorithms to sample from the set of DNA motifs matching a desired value of this most fundamental statistic.

We demonstrate their use by analyzing the informational Gini coefficient (IGC) of TF-binding motifs. Assuming that transcription factor binding motifs require a certain amount of information in order to effectively address their regulated genes, it is an open question how this information should be distributed among the positions of the motif.

**Fig. 1:**
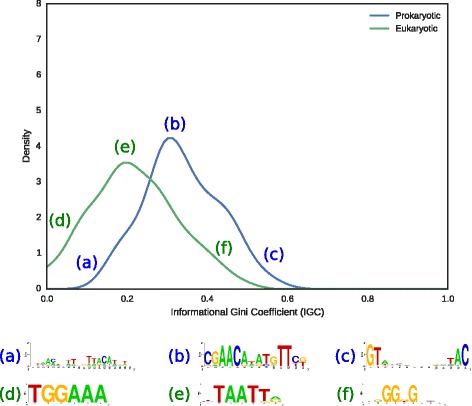
Distribution of IGC values. For prokaryotic and eukaryotic motif collections, the distribution of IGC is approximated by kernel density estimation. For each collection, the minimum, modal and maximum elements are depicted in sequence logos. Prokaryotic motifs: (*a*) OmpR, (*b*) LexA, (*c*) NtaC. Eukaryotic motifs: (*d*) NFAT, (*e*) REPO, (*f*) Macho-1

Researchers have long noted disparity in the degree of conservation between the columns of prokaryotic transcription factor binding motifs [27]. A theoretical rationale for this disparity has been proposed based on the observation of sine wave-like patterns in motifs bound by multimeric transcription factors. Regions bound through direct readout by each TF monomer require a higher degree of conservation than “spacer” regions involved primarily in backbone contacts, leading to wave-like differential patterns of information content in collections of aligned sites [28–30]. The variability in spacing between the monomer binding sites of different TFs (illustrated in Fig. 1) complicates the analysis of such patterns and the evaluation of their statistical significance. IGC measures the degree of departure from uniformity in the distribution of positional information content across a motif, without any assumptions on the particular shape of such distribution. IGC therefore provides a formal and generic statistical framework to analyze deviations from uniformity in the positional distribution of information of biological binding motifs, such as those imposed by multimeric binding.

Importantly, the distribution of information across a motif is a global property of a motif (rather than a property of its columns or column-pairs) and, therefore, it cannot be analyzed via column-wise methods. Hence, the use of random ensembles constitutes, to our knowledge, the only means of rigorously assessing the distribution of information content in TF-binding motifs. Our results show that the degree of disparity in information content across positions, as measured by IGC, is significantly higher in transcription factor binding motifs than in null ensembles with matched IC, and that higher IGC is not consistently associated with motifs bound by multimeric TFs. This indicates that the higher unevenness in the distribution of information observed in biological motifs, as measured by IGC, is an intrinsic property of TF-binding motifs, suggesting that this statistic could be exploited as a signature of biological authenticity in applications such as motif discovery.

## Methods

### Definition of the motif sampling problem

Formally, we consider a *motif* to be a matrix of gaplessly aligned sequences. Let us fix the length *L* of the sequence (in bp) and the number of sequences *N*, and consider the set $\mathcal {M}$ of all motifs with dimensions *N*×*L*, which has 4^*NL*^ elements. The choice to consider motifs extensionally as collections of sequences, rather than intensionally (e.g. PSWMs) [5], is motivated by the fact that any model of the data other than the sequences themselves is necessarily a lossy representation whose appropriateness depends on scientific context. In the interest of providing the most generally applicable results, we do not wish to commit ourselves to any particular representation of a sequence motif. Instead we prefer to work with the sequences themselves. It is also important to note that our definition of a motif technically assumes some ordering on the sequences, whereas it is more natural in most sequence analysis applications to assume that the sequences are unordered. We opt for the above definition solely to simplify the combinatorics, and our results do not depend on a choice of ordering in any way.

A *motif statistic* is any function $f:\mathcal {M}\rightarrow \mathbb {R}$. The *motif sampling problem* is then the following: given a motif *M* and a set of motif statistics {*f*_*i*_}, sample motifs *M* ^′^ with matched values of the motif statistics, i.e. so that *f*_*i*_(*M*)=*f*_*i*_(*M* ^′^) for all *i*.

Not all sampling schemes are good solutions to this problem—consider for example the trivial algorithm which returns only *M* itself with probability 1. To exclude these trivial solutions we require that the values of the motif statistics be jointly sufficient statistics for the sampling probabilities, i.e. that the probability of sampling a given motif should depend only on its values of the motif statistics, and not on any other of its properties.

Furthermore, some motif statistics may permit only the trivial solutions *M*≡*M* ^′^ identically, whereas we are really interested in the set of motifs whose statistics are *approximately* that of *M*. We therefore consider two relaxations of the motif sampling problem. On one hand, we may require the equalities to hold only in expectation, i.e. to satisfy *f*_*i*_(*M*)=〈*f*_*i*_(*M* ^′^)〉 when *M* ^′^ is sampled according to the desired algorithm. On the other hand, we may permit a small error tolerance and require only |*f*_*i*_(*M*)−*f*_*i*_(*M* ^′^)|<*ε*_*i*_ for specified values of *ε*_*i*_.

In this work we consider several motif statistics. The first is the total positional entropy of the motif, given by:

$$H(M) = -\sum\limits_{c\in M}\sum\limits_{b \in \{A,C,G,T\}}p_{c}(b)\log_{2}(p_{c}(b))), $$

where *p*_*c*_(*b*) is the sample frequency of base *b* in column *c*.^1^

Second, we define the information content (IC) of the motif to be the difference of the prior and posterior positional entropies, i.e.:

1 $$ IC(M) = H_{prior} - H(M).  $$

This quantity can be interpreted as the reduction in uncertainty as to the identity of a nucleotide sequence of length *L*, given the knowledge that it is a functional binding site. Supposing that the genomic background is well-approximated by a uniform random mononucleotide model, the prior genomic uncertainty is 2 bits/base, and Eq. 1 reduces to:

2 $$ IC(M) = 2L - H(M).  $$

The modern framework of information theory is due to Shannon [25], and the universality of motif IC as a measure of sequence conservation was first noted by Schneider and co-workers [26] in the context of transcription factor binding motifs. Through an information-theoretical argument under certain simplifying assumptions, the latter showed that the IC of a transcription factor binding motif relates the specificity of the motif to the genome size *G* and regulon size *N* through the inequality,

$$IC(M) \geq \log_{2}\left(\frac{G}{N}\right), $$

with the bound expected to be sharp in prokaryotic TFs operating without co-factors, due to evolutionary optimality. Although the bound may not universally hold in eukaryotic binding motifs due to the presence of co-factors and enhancers, IC has nevertheless become a basic statistic of interest in the bioinformatics of transcriptional regulation and molecular recognition generally [4, 31–34].

Lastly, we consider the informational Gini coefficient (IGC) of the positional information, a measure of the disparity of conservation between columns. If *c*_1_,*c*_2_,…*c*_*L*_ are the ICs of each column of *M*, sorted from least to greatest, then the IGC is given by:

$$G(M) = \frac{2\sum_{j=1}^{L} j c_{j}}{L\sum_{j=1}^{L}c_{j}} - \frac{L+1}{L}. $$

Geometrically, the Gini coefficient may be interpreted as the area of the deficit between the cumulative distribution function of a given distribution and that of a uniform distribution over the same support. It ranges between zero in the case of a uniform distribution, and unity in the case of a degenerate distribution [35].

On account of the centrality of IC in quantifying conservation in sequence motifs, we now turn to the problem of sampling motifs according to a specified value of that statistic.

### Maximum entropy approach

#### Maximum entropy distributions

The problem of sampling motifs from a distribution whose desired value of entropy is fixed in expectation can be approached through the principle of maximum entropy (MaxEnt) [36], a fully general probabilistic modeling technique only recently applied to nucleic acid motifs [37, 38]. Supposing a random variable of interest *X* with unknown distribution but the following observable constraints:

3 $$ y_{i} = \langle f_{i}(X)\rangle, i\in \{1,\ldots,n\}  $$

the probability distribution over *X* with maximum entropy subject to these constraints is given by:

4 $$  P(x) = \frac{1}{Z}\exp\left(-\sum\limits_{i=1}^{n}\lambda_{i}f_{i}(x)\right),  $$

where the conjugate variables *λ*_1_,…,*λ*_*n*_ must be tuned to match the expected values *y*_1_,…,*y*_*n*_, and the partition function *Z* ensures normalization. Distributions of this form are maximally unassuming in the specific sense that no other distribution satisfying the constraints of Eq. 3 can have greater entropy.

In this application, we choose the maximum entropy distribution over the set of motifs of given dimension, subject to a constraint on the expected value of the motif entropy itself. In practice one may consider constraining the IC instead, but this is equivalent to constraining entropy on account to of the definition of IC in Eq. 2. The resulting density takes the form of a Boltzmann distribution with Shannon entropy in place of energy:

5 $$  P(M) = \frac{1}{Z}e^{-\lambda H(M)},  $$

where,

6 $$ Z=\sum\limits_{M\in\mathcal{M}}e^{-\lambda H(M)}  $$

is the partition function and *λ* is tuned so that 〈*H*(*M*)〉_*P*_, the expected value of entropy when *M* is distributed according to *P*(*M*), attains the desired value.

This distribution satisfies the stated requirements of the first formulation of the motif sampling problem. The average value of the motif IC is respected, and the probability of a motif depends only on its IC and not on any of its other properties.

The description of a probability distribution, however, does not by itself suffice to sample from it. We complete this section by introducing an exact algorithm for effective sampling from distributions of this type.

#### Maximum entropy sampling

In principle, any discrete MaxEnt distribution can be sampled rather mechanically: one need merely compute the partition function in Eq. 6 and employ the inverse transform method to obtain samples. In this application, however, *Z* contains 4^(*NL*)^ terms and will be impossible to enumerate directly for all but the smallest dimensions.

Distributions with intractable normalization constants are often approachable via Markov Chain Monte Carlo techniques [39–41]. In the absence of an explicit convergence criterion, however, the practitioner must rely on heuristics or visual inspection to gauge approximate stationarity of the chain. For this reason, we are motivated to derive an exact algorithm instead.

Let us first consider the set of motifs of dimension *N*×1, i.e. those consisting of a single column of length *N*. The probability assigned to each motif in that set must depend only on its entropy, and hence only on the frequencies of each nucleotide. This observation permits us to partition the set of motifs into a smaller set of equivalence classes defined by equality of entropy. To understand this equivalence relation, we can define a counting function *κ* that takes each motif to the vector of its nucleotide counts 〈*n*_*A*_,*n*_*C*_,*n*_*G*_,*n*_*T*_〉. Two motifs are then equivalent with respect to entropy if their count vectors are permutations of one another. To efficiently compute the partition function, then, it suffices to compute the entropies of each unique vector of counts (up to permutation) and weight them by the cardinality of their equivalence classes.

In detail, the entropy associated with a count tuple $\vec {n}$ is:

$$H(\vec{n}) = -\sum\limits_{{b \in \{A, C, G, T\}}}\frac{n_{{b}}}{N}\log(\frac{n_{{b}}}{N}), $$

Next, define a weight function *w* by,



where $\mathcal {M}$ is the set of *N*×1 motifs and ∼ denotes equivalence under permutation. Informally, *w* counts the number of motifs that map to each distinct tuple. For ease of reference, when we consider equivalence classes of count tuples we will take the first element of the class, sorted lexicographically, as its distinguished representative. Now it is possible to obtain a convenient closed form. To do so, we first define:



Informally, *m* counts the *multiplicities* of each element of the count tuple. Then we have:

7 $$ {} w(\vec{n}) = \left(\frac{4!}{\prod\limits_{{{b \in \{A, C, G, T\}}}}m(\vec{n})_{{b}}!}\right) \left(\frac{N!}{\prod\limits_{{{b \in \{A, C, G, T\}}}}n_{{b}}{!}}\right).  $$

This formula has an elementary interpretation: the first term counts the permutation-equivalent count vectors, and the second term counts the number of motifs associated with each count vector.

At end, we need only compute the sum:

8 $$  Z_{c}(\lambda) = \sum\limits_{\vec{n}\in \mathcal{N}/\sim}w(\vec{n})e^{-\lambda H(\vec{n})},  $$

where $\mathcal {N}/\sim $ is the quotient of the set of count vectors by permutation equivalence, $\vec {n}$ ranges over the distinguished representatives of all equivalence classes, and we write *Z*_*c*_ to remind that this is the partition function for a single column. In this way we can reduce the sum to a tractable number of terms that can be computed exactly. For *N*=200, for example, there are fewer than 6×10^4^ terms in the sum over equivalence classes, as opposed to approximately 2×10^120^ in the full sum.

Finally, having treated the single-column case, let us now consider multiple columns. Because positional entropy is additive with respect to columns, we can rewrite the expression in Eq. 5 as:

$$\begin{array}{*{20}l} P(M) &= \frac{1}{{Z_{c}^{L}}}e^{-\lambda \sum_{j=1}^{L}H(M_{j})}\\ &= \frac{1}{Z}\prod\limits_{j=1}^{L}e^{-\lambda H(M_{j})},\\ \end{array} $$

where *M*_*j*_ denotes the *j*th column of *M*, and $Z\equiv {Z_{c}^{L}}$. The MaxEnt distribution over motifs therefore factors into the product of MaxEnt distributions for the columns. Sampling a multiple-column motif is then only a matter of sampling *L* columns independently and adjoining them.

The expected entropy of a motif as a function of *λ* is given by:

9 $$  \langle H(M) \rangle_{\lambda} = L \sum\limits_{\vec{n}\in \mathcal{N}/\sim}H(\vec{n})\frac{w(\vec{n})e^{-\lambda H(\vec{n})}}{Z(\lambda)}.  $$

The appropriate setting of *λ* for a desired value of entropy *H* ^∗^ can be found by solving:

10 $$  \langle H(M) \rangle_{\lambda} - H^{*} = 0  $$

via standard root-finding algorithms [42].

#### Runtime analysis

The runtime of the algorithm described in the previous section is dominated by the task of estimating the parameter *λ*, which requires evaluation of the weight function in Eq. 7 for every count vector as well as computation of the sum in Eq. 8. Both of these tasks are linear in the size of the count vector quotient $\mathcal {N}/\sim $ defined in Eq. 8. The size of the count vector quotient is just the number of integer partitions of *N* having at most 4 parts. This quantity has a convenient closed form [43],

$${\begin{aligned} |\mathcal{N}/\sim| = \text{round}\left(\frac{(N+4)^{3} + 3(N+4)^{2} - 9(N+4)((N+4)\ \text{mod}\ 2)}{144}\right), \end{aligned}} $$

which is $\mathcal {O}(N^{3})$. Once *λ* has been fixed, the remaining sampling steps require $\mathcal {O}(L\log (|\mathcal {N}/\sim |))$ operations. The total runtime is therefore linear in *L* and cubic in *N*. Although the time complexity of the algorithm is polynomial in both *N* and *L*, we caution as a practical matter that runtimes may still be long when *N* is large.

### Truncated uniform approach

#### Truncated uniform distributions

While the MaxEnt distribution described in the previous section is the maximally unassuming model for motifs of a given mean IC, the user might find it more convenient in some cases to explicitly define the range of permissible IC values to sample from. We therefore consider the task of sampling uniformly from the set of all motifs having a given IC of *I*±*ε* bits. This distribution has the form:

$$P(M) =\left\{ \begin{array}{ll} \frac{1}{Z}, & |I - IC(M)| \leq \varepsilon \\ 0, & \text{otherwise} \end{array} \right. $$

where *Z* normalizes. This distribution satisfies the stated requirements of the second formulation of the motif sampling problem.

#### Truncated uniform sampling

To sample from the truncated uniform distribution on IC we propose a rejection sampling algorithm [44] that employs the MaxEnt distribution as a proposal. Fixing the desired IC *I*±*ε*, let

$$Q(M)=\frac{e^{\lambda IC(M)}}{Z_{Q}} $$

be the p.m.f. of a MaxEnt proposal distribution with mean IC *I*. (For notational convenience we will consider *Q* to be defined in terms of information content rather than entropy; the two formulations are equivalent up to a sign change and a constant.)

Now let,

$$P(M)=\frac{[|IC(M) - I| < \varepsilon]}{Z_{P}} $$

be the p.m.f. of the target distribution. If there exists a positive constant *C* such that $\frac {P(M)}{CQ(M)}\leq 1$ for all *M*, then it is possible to sample from *P* by drawing a sample *M* from *Q* and a uniform random variate *r* from *U*[0,1], accepting *M* only if $\frac {P(M)}{CQ(M)}\leq r$.

Although both *P* and *Q* contain intractable normalization constants, we can nevertheless write:

$$\begin{array}{*{20}l} \frac{P(M)}{Q(M)} =& \frac{\hat{P}(M)/Z_{P}}{\hat{Q}(M)/Z_{Q}}\\ =& e^{-\lambda IC(M)}\frac{Z_{Q}}{Z_{P}},\\ \end{array} $$

and absorb the ratio $\frac {Z_{Q}}{Z_{P}}$ into the constant *C*. From then on, we may simply consider the ratio of the unnormalized densities,

$$\frac{\hat{P}(M)}{\hat{Q}(M)} = \frac{1}{e^{\lambda IC(M)}}, $$

which is maximized for *I* *C*(*M*)=*I*−*ε*≡*I*_*min*_. Therefore we set $C=e^{-\lambda I_{min}}\phantom {\dot {i}\!}$. Altogether, the acceptance ratio *AR* for a motif *M* drawn from *Q* is given by:

$$AR(M) = e^{\lambda(I_{min} - IC(M))} $$

In particular, the acceptance ratio is never less than *e* ^−2*λε*^.

The total time required to draw one motif from *P* therefore goes as the product of *T*_*Q*_, the time required to sample from *Q*, and $\langle \frac {1}{AR}\rangle _{Q}$, the mean number of trials required in order to accept a sample. If *ε* is small, then we have $T_{Q}\propto \frac {1}{\varepsilon }$, whereas the mean number of proposals will be bounded above by *e* ^2*λε*^. Hence the CPU time will go approximately as $\frac {e^{2\lambda \varepsilon }}{\varepsilon }$, which is minimized for

11 $$ \varepsilon=\frac{1}{2\lambda}.  $$

Therefore, although it is possible to set *ε* to any desired value, an approximately optimal error tolerance follows directly from the runtime analysis.

### Parametric bootstrapping

For any motif *M* and motif statistic *f*, we may compute the bootstrap percentile of *y*=*f*(*M*) in the following way. First we compute *I*=*I* *C*(*M*), then sample $M^{\prime }_{1},\ldots M'_{n} \stackrel {i.i.d.}{\sim } P(M'|I)$, for *P* the parametric density of our choice (MaxEnt or TU). Setting $y^{\prime }_{i} = f(M'_{i})$ for each *i*, the *bootstrap percentile* of *y* is given by,

$$p_{BS}(f,M) = \frac{|\{y'_{i} | y' < y\}|}{n}, $$

or the fraction of observed bootstrap replicates having a value of the motif statistic *f* less than *f*(*M*) [45].

### Information content *p*-value calculations

MaxEnt distributions subject to mean entropy may be exploited in order to estimate the *p*-value of a motif’s information content. In this application, the task is to determine the probability of observing a motif of given dimension with IC of at least *I* bits by chance. Although exact algorithms for this problem have proven elusive, [46, 47], we suggest an application of the MaxEnt framework to this problem, yielding an importance sampling estimate that may be bounded analytically, approximated by moment matching, or estimated efficiently through a Monte Carlo importance sampling estimate.

Formally, the *p*-value of the information content *I* of a motif *M* is given by,

12 $$  p_{val} = \frac{1}{Z_{P}}\sum\limits_{M\in \mathcal{M}}[IC(M) > I],  $$

where *Z*_*P*_=4^*N*×*L*^. Let $Q(M)=\frac {1}{Z_{Q}}e^{\lambda IC(M)}$ be the MaxEnt distribution subject to mean IC such that the expected IC under *Q* is *I*. Then Eq. 12 can be recast as an importance sampling estimate,

13 $$  p_{val} = \frac{Z_{Q}}{Z_{P}}\langle [IC(M) > I] e^{-\lambda IC(M)}\rangle_{Q}.  $$

with the expectation taken according to *Q*.

#### Analytic upper bounds

We can obtain an upper bound on the *p*-value by noting that the expression inside the expectation, [*I* *C*(*M*)>*I*]*e* ^−*λIC*(*M*)^, will never be greater than *e* ^−*λI*^. Under the assumption that the induced distribution on IC is symmetric about *I*, we can reduce this bound further by a factor of two since half of the samples will not contribute to the sum, yielding:

14 $$ p_{val} \leq \frac{Z_{Q}}{Z_{P}} \left[\frac{1}{2}e^{-\lambda I}\right].  $$

#### Moment-matching estimates

It is also possible to estimate the expectation analytically. The distribution *Q*(*M*) over motifs induces a distribution *I* *C*(*M*) on information content which is approximately normal. The mean of that distribution is just *I*, by construction. The variance *σ* ^2^ can be computed exactly by summing over count vectors in the fashion of Eq. 9. The expectation can therefore be recast as the integral,

15 $$  \langle [IC(M) > I] e^{-\lambda IC(M)}\rangle_{Q} \approx \int_{I}^{\infty}e^{-\lambda x}\phi(x;I, \sigma^{2})\ dx,  $$

where *ϕ* is the normal probality density function. This integral has an exact solution [48], allowing the *p*-value to be written altogether as:

16 $$  p_{val} \approx \frac{Z_{Q}}{Z_{P}}\left[\frac{1}{2} e^{-\lambda I}\text{erfcx}\left(\frac{\lambda\sigma}{\sqrt{2}}\right)\right],  $$

where erfcx is the scaled complementary error function [49]. In fact, Eq. 16 can be read as Eq. 14 adjusted by a factor of $\text {erfcx}\left (\frac {\lambda \sigma }{\sqrt {2}}\right)$, which is bounded in the unit interval for positive arguments and captures the effect of the dispersion of the IC values from *Q* about *I*.

#### Importance sampling

Finally, we note that the accuracy of these methods may be checked by simply taking an importance sampling estimate, drawing *M*_1_,*M*_2_,…*M*_*n*_∼*i*.*i*.*d*.*Q* and replacing the expectation in Eq. 15 with a sample mean:

17 $$  p_{val} \approx \frac{Z_{Q}}{Z_{P}}\left[\frac{1}{n}\sum\limits_{i=1}^{n}[IC(M_{i}) > I] e^{-\lambda IC(M_{i})}\right].  $$

#### Conversion to E-values

To convert from *p*-values to E-values, which are more common in the context of motif discovery, we simply multiply the *p*-value by the number of alignments possible under the given data model (e.g. OOPS, ZOOPS, ANR, &c.) [46], various results for which are collected in [50].

### GC-content adjustment

As Eq. 4 suggests, the MaxEnt framework permits the incorporation of as many mean constraints as the modeler sees fit. We have chosen to focus on information content as a universal statistic of sequence specificity, but other constraints may sometimes prove desirable as well. In the context of transcription factor binding motifs, which draw their instances from typically AT-rich promoter regions, one may prefer to control for GC-content as well as IC.

Formally, this amounts to finding parameters *λ* and *μ* so that the system:

$$\begin{array}{*{20}l} P(M) &= \frac{1}{Z}\exp\left(\lambda IC(M) + \mu GC(M))\right),\\ \langle IC(M)\rangle &= I,\\ \langle GC(M)\rangle &= G, \end{array} $$

is satisfied for desired IC *I* and GC *G*. In general, a system with multiple constraints might be solved by generic optimization techniques such as stochastic gradient descent. In this case, however, we can exploit the fact that the computation of *λ* is independent of GC in order to first fix mean IC, and then fix mean GC conditional on mean IC. In this way, we preserve both the exactness of the parameters and the efficiency of the sampling algorithm.

In particular, the expected GC-content of a column is given by:

18 $$  \langle \%GC \rangle = \sum\limits_{\vec{n}}P(\vec{n})\left(\sum\limits_{g\in\vec{n}}g\cdot \frac{u(g|\vec{n})e^{-\mu g}}{Z(\vec{n},\mu)}\right),  $$

where $g \in \vec {n}$ ranges over the possible GC-contents derivable from the count vector $\vec {n}$, $u(g|\vec {n})$ is the base probability of selecting a GC content of *g* from $\vec {n}$, (i.e. if *μ* were 0), and $Z(\vec {n},\mu) = \sum _{{g\in \vec {n}}}u(g|\vec {n})e^{-\mu g}$ is the partition function over GC levels for a given count vector $\vec {n}$. Finding the value of *μ* that yields the desired mean GC-content is a one-dimensional root-finding problem that may be solved on analogy with Eq. 10.

We caution that IC and %GC are not independent: it is impossible, for example, for a column to be fully conserved while maintaining a %GC of 0.5. In general, the minimum (respectively, maximum) %GC is given by $\sum \limits _{\vec {n}}\mathrm {P}(\vec {n})\min \{g|g\in \vec {n}\}$, (respectively, max). If desired %GC exceeds these bounds, then there will be no values of *λ* and *μ* that simultaneously satisfy the constraints. In our implementation, we check for existence of the solution and warn the user if necessary.

### Data curation

In our analysis we examined naturally occurring, experimentally validated transcription factor binding motifs from both prokaryotic and eukaroytic organisms. Prokaryotic motifs were accessed from the CollecTF, MtbRegList, RegTransBase, RegulonDB, DBTBS, and CoryneRegNet databases [51–56]. Eukaryotic motifs were accessed from the JASPAR vertebrate database [57]. Motifs that had fewer than ten sites, inconsistent lengths, fewer than five bits of IC, or which were derived from SELEX were excluded. Motifs with more than 200 sites were randomly subsampled without replacement to a final depth of 200 sites.

Provided that this down-sampling is performed at random, the only potential concern is small sample bias. The sample-size correction for IC is $\mathcal {O}(\frac {1}{N})$ [58], amounting to less than 0.01 bits per position when *N*=200. Since IGC is a function of the positional IC values of a motif, its own small sample size correction is also negligible. Statistics for down-sampled motifs can therefore be confidently imputed to the originals.

Summary statistics are given in Table 1 and presented graphically in an additional file [see Additional file 1].

**Table 1:** Motif summary statistics

	Prokaryotic			Eukaryotic		
	Min	Median	Max	Min	Median	Max
Length (bp)	9	17	29	4	9	26
Number of Sites	10	19	459	10	44	19,264
IC (bits)	6.10	13.05	21.57	5	12.70	33.48
IGC	0.16	0.33	0.56	0	0.21	0.51
Number of Motifs	63			424		

Minimum, median and maximum statistics are presented separately for prokaryotic and eukaryotic motifs. Maximum statistics for number of sites refer to values prior to subsampling

### Hardware

All timing simulations were performed on a desktop workstation, using a single 3.3 GHz Intel Xeon core with an 8MB cache, and 16 GB DDR RAM.

### Software

Algorithms described in this study are implemented in a Python 2.7 library available at github.com/poneill/formosa.

## Results

### Validation and performance

**Fig. 2:**
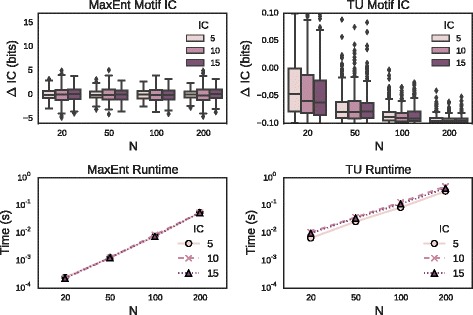
Algorithm Validation and Runtime Study. (*Top Row*) Sampled motif IC for MaxEnt and Truncated Uniform algorithms, zeroed by desired IC. For a motif of length 10 and number of sites varying from 20 to 200, each algorithm was called 100 times each with a target IC value of 5, 10 or 15 bits. (*Bottom Row*) Runtime per motif, using same parameters as above. Times were averaged over three trials

We begin by validating the output of both algorithms. Figure 2 shows the distribution of IC of motifs sampled via both the MaxEnt and TU distributions (top left and top right, respectively) under various parameter settings. In particular, the length of the motif was fixed at 10 bp; the number of sites in the motif *N* varied from 20 to 200; and the desired IC varied between 5 and 15 bits with a tolerance of ±0.1 bits for the TU distribution. In each case we find good agreement between the desired and obtained IC values.

In the lower panels we display CPU times required to perform the sampling. Depicted for each parameter setting is the mean time required to sample a motif when requesting 100 random motif variates, with each call averaged over three trials. In all cases, the required time is less than 1s per motif.

**Fig. 3:**
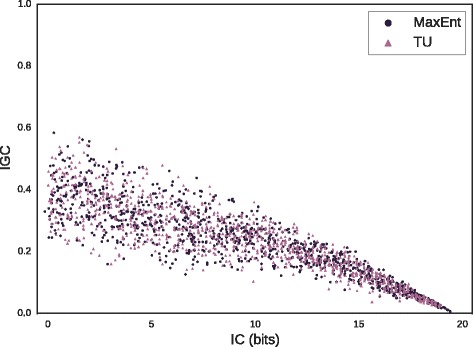
Conditional distribution of IGC given IC. For *L*=10, *N*=50 and IC varying uniformly from 0.1 to 19 bits, 100 motifs were sampled from the MaxEnt and TU distributions for each IC value, and IGC plotted against IC

To compare the distributions on IGC induced by the MaxEnt and TU distributions, we fixed motif parameters *L*=10 and *N*=50 and then sampled from each distribution, with desired IC increasing from 0.1 to 1.9 bits over 100 steps. Results, shown in Fig. 3, show that the MaxEnt and TU distributions have quite similar distributions of IGC conditional on IC. In agreement with intuition, IGC approaches 0 as IC approaches a maximal value of 2 bits per column, as all columns become fully conserved. As IC decreases (right to left), mean IGC increases. To assess whether the distribution of IGC conditional on IC differed between the two algorithms, MaxEnt and TURS IGC values for each IC step were compared via Kruskal-Wallis test [59]. To account for multiple hypothesis testing, the significance level was adjusted via the Benjamini-Hochberg procedure [60] to hold the false discovery rate (FDR) to 5 %. To within the limits of statistical power, there is no detectable difference in the distribution of IGC conditional on IC between the two algorithms (Kruskal-Wallis test with FDR correction *p*>0.05).

### IGC in biological motifs

**Fig. 4:**
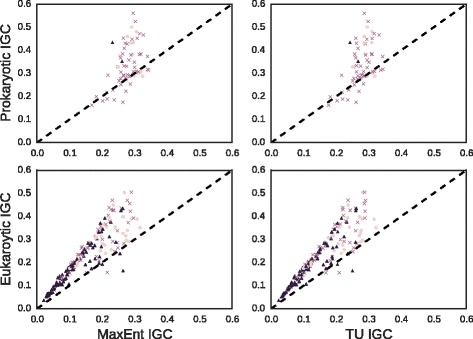
Distribution of IGC in Biological vs. Synthetic Motifs. For both prokaryotic and eukaryotic motifs (*top and bottom row, resp.*), observed IGC is plotted on Y-axis against mean sampled IGC given IC on X-axis under MaxEnt and TU distributions (*left and right columns, resp.*) Colors and markers denote motif structure (*dark circle: inverted repeat; medium cross: direct repeat; light triangle: monomer*). The identity function y=x is plotted as a *dashed line* to guide the eye, and the axes have been truncated as an aid to legibility

We then explore how the IGCs of biological motifs compare to those from matched synthetic motifs. We separately considered the prokaryotic and eukaryotic collections of naturally-occuring, experimentally validated transcription factor binding motifs. For each motif we generated 100 matched motifs according to both the MaxEnt and TU distributions, then compared the observed IGC to the mean of the synthetic motifs. To explore the possibility of a relationship between IGC and basic motif structure, we classified each motif as an inverted repeat, direct repeat or monomer. The results are shown in Fig. 4.

For prokaryotic motifs, the Pearson correlations between biological and synthetic IGCs are statistically significant for both the MaxEnt and TU distributions (*r*=.50, *p*<10^−4^, and *r*=.52, *p*<10^−4^, respectively). For eukaryotic motifs, the correlations are even higher (*r*=.92, *p*<10^−93^, and *r*=.93, *p*<10^−96^, respectively). It is clear that our model is capturing some fraction of the variation in IGC. We also observe, though, that the biological values of IGC are typically higher. This is especially true of eukaryotic motifs. The basic motif structure is indicated in the style of the marker, showing no clear relationship between structure and IGC. For reference, the IC values of the biological motifs are also shown against the mean IC values of their matched replicates. These plots indicate good agreement in all cases.

**Fig. 5:**
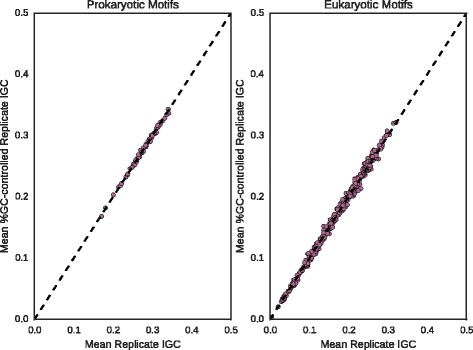
IGC of MaxEnt replicates vs. %GC-controlled MaxEnt replicates. For each transcription factor binding motif, the mean IGC of an ensemble of MaxEnt replicates is compared to the mean IGC of a second ensemble additionally matched for %GC. For each plot, Pearson *r*>0.99. The identity function y=x is plotted as a *dashed line* to guide the eye, and the axes have been truncated as an aid to legibility

To ensure that the effect observed in Fig. 4 was not due merely to systematic differences in %GC between transcription factor binding motifs and random controls, we generated for each motif an ensemble of randomised motifs matching mean IC only, and a second ensemble matching mean IC and mean %GC. We then compared their distributions of IGC in order to test for possible bias in %GC-controlled motifs towards higher IGC values. In Fig. 5, we compare mean IGC for each pair of ensembles, finding excellent agreement (Pearson *r*>.99).

### IGC as a signature of biological origin

Noting that the IGCs of biological motifs are generally higher than those of synthetic motifs with matching IC, we consider next whether this phenomenon could be employed as a predictor of biological origin. For each biological motif we compute the bootstrap percentile of its IGC value from a sample of 100 MaxEnt motifs. These percentiles are typically high; in the prokaryotic collection, for example, 14 of 63 motifs were found to have IGCs greater than all 100 of their bootstrap replicates, whereas the number of such motifs expected by chance is less than one.

**Fig. 6:**
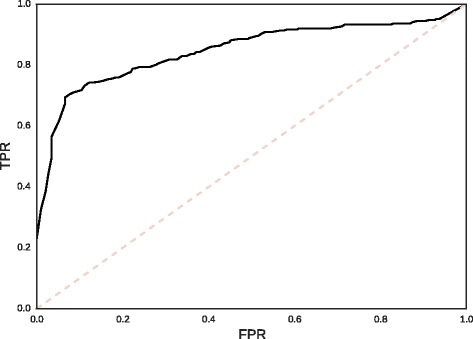
ROC curve for IGC as a predictor of biological origin. For all biolgical motifs in this study, the IGC bootstrap percentile was estimated using a sample of 100 MaxEnt motifs each, and compared to the IGC bootstrap percentiles of an equal number of MaxEnt negative controls. AUC ≈0.85

To construct a negative set, one MaxEnt motif was sampled from each biological motif, and IGC bootstrap percentiles were computed similarly. Figure (6) depicts the resulting receiver-operating characteristic (ROC) curve for IGC percentile as a predictor for distinguishing biological from synthetic motifs. An ROC curve can be used to describe the performance of a binary classifier with a tunable threshold by comparing the true positive rate (TPR) to the false positive rate (FPR) over all possible values of the threshold. A perfect classifier can simultaneously achieve a TPR of 100 % and a FPR of 0 %, whereas for an arbitrary random classifier the TPR will generally be equal to the FPR for any threshold value. By integrating the ROC curve, one obtains the “area under curve” (AUC) statistic, which will vary between unity for a perfect classifier, and 0.5 for a random classifier [61]. In this case, the AUC statistic is approximately 0.85.

### Motif IC *p*-values

**Fig. 7:**
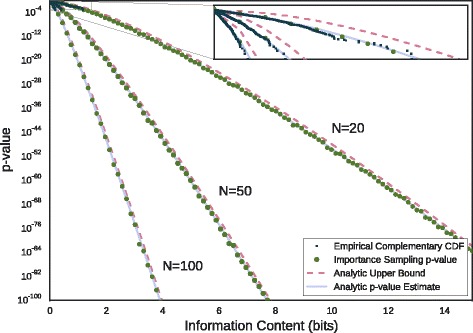
IC *p*-value estimates. *Upper* bounds and analytic estimates for motif IC *p*-values are shown for the parameter values L = 10, *N* = 20,50,100. Also shown is the empirical complementary cumulative distribution function of 10 ^3^ randomly sampled motifs, as well as *p*-values derived from importance sampling. Inset: Same data

Turning to the problem of estimating motif IC *p*-values with the MaxEnt framework, we validate our methods in Fig. 7. Fixing *L*=10 and varying *N* between 20, 50 and 100, we estimate *p*-values according to the bounding and moment-matching approaches discussed above (Eq. 14 and Eq. 15, resp.) for IC ranging between 0 and 15 bits. For further comparison, the cumulative density function (CDF) of the null IC distribution for each value of *N* was estimated empirically from 10^3^ samples each. For IC values beyond the detection limit of direct sampling, *p*-values were computed by importance sampling (Eq. 17), with 10^3^ samples per data point.

The inset plot shows good agreement between both methods and the empirical complementary CDF, up to the event detection limit of 10^−3^. Beyond this limit, the methods continue to agree with the importance sampling *p*-values past 10^−100^ with the upper bound typically holding to within a single factor of ten. In fact these trends persist over the full domain of IC, though we have truncated the y-axis for clarity.

### False-positive identification in motif-finding settings

**Fig. 8:**
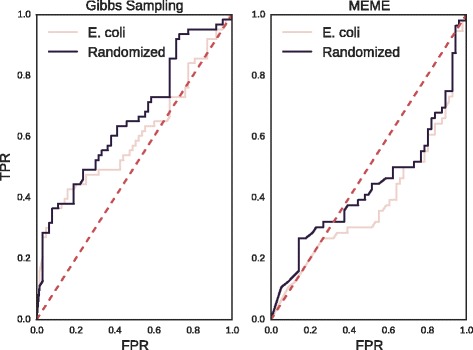
IGC percentile ROC curves in Gibbs sampling and MEME. Prokaryotic motifs are distinguished from synthetic motifs on the basis of IGC percentile. Synthetic motifs were derived either from Gibbs sampling (*left*) or MEME (*right*), performing motif discovery on either *E. coli* coding sequences or a random mononucleotide model. For Gibbs sampling, AUC values are approximately 0.60 for *E. coli* sequences and 0.65 for synthetic sequences. For MEME, AUC values are approximately 0.38 for *E. coli* sequences and 0.44 for synthetic sequences

In the previous section, we introduced methods to estimate the *p*-value of a given motif. Conversely, these methods can also be applied to the problem of estimating the length of a collection of sequences to be input into a motif-finding algorithm in order to yield one motif of given dimensions and IC. This allows us to benchmark the advantages of the IGC percentile as a potential filter for false positives in motif-finding algorithms. To this end, we considered another classification task similar to that of Fig. 6, but in which the negative set is drawn from motif-finding algorithms rather than from MaxEnt distributions. For each motif in our collection we constructed random sets of sequences drawn either from a mononucleotide model or from the coding sequences of the *Escherichia coli* K12 MG1655 genome [62]. Eukaryotic motifs were excluded due to the difficulty of generating synthetic false positives of such characteristically large size. *E. coli* genomic sequences were restricted to coding regions in order to preclude the possibility of recovering a genuine transcription factor binding motif. One motif of equivalent dimensions was then extracted from each collection using a Gibbs sampling approach [63] under the assumption of a one-occurence-per-sequence model. Considering these motifs as false positives, we then attempted to discriminate them from true TF binding motifs as in Fig. 6, constructing 1000 MaxEnt replicates for each and measuring their IGC percentiles. AUC values range from 0.60 for *E. coli* sequences to 0.65 for synthetic sequences. We note in particular that for motifs drawn from *E. coli* sequences, classifying power is greatest for high IGC percentiles and falls off to approximately random chance as the threshold decreases, whereas for random sequences the classifying power is approximately constant with respect to threshold. Results are shown in the left panel of Fig. 8.

We next performed an analogous experiment with synthetic motifs drawn this time from the MEME algorithm [14]. Due to the computationally intensive nature of MEME, seven large prokaryotic motifs timed out and were excluded from further analysis, leaving 56 motifs in total. The results, shown in the right panel of Fig. 8, indicate that the IGC percentile criterion yields AUC values of less than 0.5 when used to discriminate TF binding motifs from false positives produced by MEME.

**Fig. 9:**
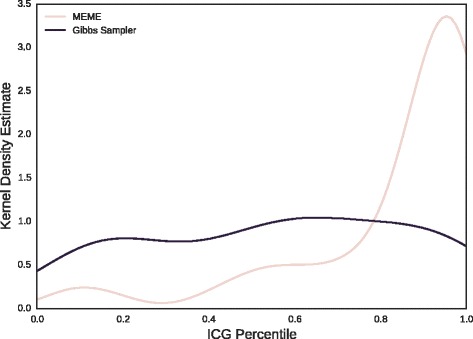
Distribution of IGC percentile for synthetic motifs. Kernel density estimates of the distributions of IGC percentiles are shown for synthetic motifs derived from random sequences via Gibbs sampling and MEME (following Fig. 8)

To explore this last unexpected result, the distribution of IGC percentiles for synthetic motifs drawn from randomized sequences through Gibbs sampling or MEME (following Fig. 8) are compared in Fig. 9. There we see that while the percentile distribution is roughly uniform for motifs found through Gibbs sampling, there is a pronounced peak in high IGC percentiles for motifs found through MEME, indicating a bias in MEME towards high IGC, relative to expectation.

## Discussion

In this study we present a general framework for analyzing the properties of biological sequence motifs. This method arises from the general problem that, given a motif with some seemingly unusual feature, we often wish to know whether that feature is remarkable in the statistical sense. The question then arises of what to compare the given motif *to*. The most natural null distribution is the set of all motifs with comparable IC, but it is not obvious how to obtain them.

Consider a motif of modest dimensions *L*=10 and *N*=20 with 10 bits IC, for example. There are 4^200^≈2×10^120^ motifs of this size, of which we can calculate that approximately 2×10^78^ have 10±0.1 bits IC. The probability of obtaining one such motif by sampling uniformly at random is therefore on the order of 10^−42^, implying runtimes longer the timescale of the universe. Alternately, one could construct high IC motifs heuristically, but there is no guarantee that the resulting replicates would satisfy the requirements of the motif sampling problem as stated. To our knowledge, prior to this study the problem of sampling a motif with a desired quantity of IC has never been addressed.

The algorithms developed here allow users to sample from the space of all motifs of given dimension so that the generated variates have *I* bits of IC on average (for MaxEnt) or all have IC within *ε* bits of *I* (for TU).

### Validation and performance

While validating the methods, we noticed that although the agreement between specified and sampled IC in Fig. 2 was generally good, the Truncated Uniform distribution was biased towards the lower endpoint of the IC interval, especially for large *N*. This is an expected consequence of the fact that the base density of motifs is approximately inversely exponential in IC. Thus a distribution which assigns equal probability mass to all motifs in a given range of IC will be biased towards the lower end of the interval simply because there are more motifs there to be sampled.

Due to the fact that the algorithm for the TU distribution employs a MaxEnt sampler as a proposal for a rejection sampling scheme, TU runtimes are never lower than MaxEnt runtimes for the same parameters. Specifically, the TU runtimes go inversely with the acceptance ratio. For ease of comparison between parameter settings, we set $\varepsilon =\frac {1}{10}$ throughout and found acceptance ratios typically not less than 10^−2^. This will naturally degrade, however, as *ε*→0. The user who wishes to maximize the acceptance ratio may set *ε* according to the heuristic we describe in Eq. 11.

When we examined the relationship between IC and ICG generated by the MaxEnt and TU distributions in Fig. 3, we saw that mean IGC increases as IC decreases, a phenomenon due simply to the greater number of ways to satisfy the required IC with high disparity of conservation between columns, relative to the number of low-disparity solutions. The variance also increases with decreasing IC, indicating that low-IC motifs admit a wider variety of solutions in terms of columnar conservation.

### IGC in biological motifs

Comparing biological motifs to random ensembles of synthetic motifs with matched IC in Fig. 4, we find that the ensemble statistics account for some fraction of the observed variation, leading to statistically significant correlations in both the prokaryotic and eukaryotic collections. On the other hand, we also find that biological motifs typically show higher IGC than would be expected by chance. Moreover, this phenomenon is independent of the choice of null distribution.

The conventional explanation for the inequal distribution of information content in TF-binding motifs is based in the informational footprint associated with sites bound by TFs in multimeric conformation (typically as homodimers) that is observed in a large fraction of prokaryotic TFs. The combination of low specificity binding at spacer regions with highly specific readout at monomer-binding sites leads to a characteristic wave-like pattern in the distribution of information content on aligned binding sites. We do not, however, observe any clear trend between motif structure and IGC. Furthermore, the pattern of greater than expected IGC persists in eukaryotic motifs, where the large majority of transcription factors are monomeric [64]. Comparisons between biological motifs and the synthetic replicates used to assess IGC differences show good agreement in IC. Hence the observed discrepancy in IGC cannot be attributed to any discrepancy in IC introduced by the sampling process, nor to multimeric binding mode, and comparison of IGC values for MaxEnt ensembles with and without controlling for %GC in Fig. 5 confirms that this deviation is not caused by %GC bias in TF-binding motifs. This suggests that the uneven pattern of information encoding seen in TF-binding motifs is the result of other biochemical or informational constraints on TF-binding site evolution.

DNA accessibility on the axis of binding impacts the ability of proteins to specifically recognize individual DNA bases, both across grooves and between them [65, 66], providing a potential explanation for the deviation in IC distribution observed in TF-binding motifs, independent of binding mode. The uneven encoding of binding information, however, can also provide advantages from an informational point of view. Theoretical analyses have shown that the short length of TF-binding sites enhances their mutational robustness [8]. Deviation from uniformity in the distribution of information across a TF-binding motif effectively translates into a form of site compression for mutational purposes, with conserved positions acting as hubs in the space of viable TF-binding sites, and hence provides an informational basis for the observation that TF-binding sites are both more mutationally robust and evolvable than one would expect by chance in sequences of similar length [67].

### IGC as a signature of biological origin

As an illustration of possible applications, we have shown that the bootstrap percentile of IGC is an effective feature for discriminating biological motifs from synthetic motifs of comparable IC. As a proof of concept, we consider the task of distinguishing biological motifs from IC-matched MaxEnt control motifs. We employ MaxEnt distributions as negative controls precisely because they are least assuming distributions that satisfy the expected ICs. Using only IGC as a predictor to identify the biological motifs, we find that such a classifier achieves an AUC of approximately 0.85. Crucially, this feature is orthogonal to IC, and can therefore provide an independent signal of biological authenticity in transcription factor binding motifs for ensemble classifiers. For example, consider the problem of prioritizing further research into putative motifs found through a motif discovery application. Among motifs with equal E-values, there is no a priori reason to prefer one to another. By ranking the motifs according to their IGC bootstrap percentiles, however, one can prioritize a random biological motif before a random false positive with about 85 % probability. This scenario suggests that the IGC is a fairly robust signature of biological provenance in transcription factor binding motifs, and may therefore prove useful in the context of motif discovery as an adjuvant to conventional E-value-based methods of assessing significance.

### Motif *p*-values

Next we turn to the exploration of motif *p*-value estimates depicted in Fig. 7. The problem of motif *p*-value estimation is central to the task of motif discovery, helping workers to distinguish between motifs likely to possess biological relevance and spurious results due to chance. The problem of statistical significance is especially acute in this setting since the search, being NP-hard, takes place over a necessarily combinatorial space of possible motifs [68]. Accordingly, motif *p*-value calculation algorithms tend to invoke the machinery of generating functions [69, 70], dynamic programming [68], or related methods such as branch-and-bound algorithms implemented with fairly sophisticated data structures [71]. Of interest to us was the question of whether the MaxEnt framework could be pressed into service of the motif *p*-value problem in order to yield a conceptually simple Monte Carlo calculation or some related estimators.

Comparing our methods to simulations in Fig. 7, we find strong agreement between the upper bound of Eq. 14 and the analytic estimate of Eq. 15 on one hand, and the empirical *p*-values found by direct sampling and importance sampling on the other. The strategy of bounding the *p*-value from above is an analytic result requiring no further assumptions, and in particular holds over all possible distributions on IC which satisfy the constraint on the mean. Despite this generality, it appears to hold consistently within a factor of ten of the true *p*-value over a hundred orders of magnitude. The moment-matching estimate is more precise, but assumes that the distribution induced on IC is approximately normal. This assumption is easily granted when the dimensions of the motif are large, but even for modestly sized motifs (e.g. *L*=10,*N*=20) we found excellent agreement with empirical simulation. These estimators fall out more or less naturally from the MaxEnt framework, and illustrate its utility in rare-event estimation.

After developing this application we noticed an interesting congruence between our rejection sampling algorithm and the method of Hertz and Stormo [46]; the latter approached the *p*-value problem via large deviations theory and the method of exponential tilting, whereas we were motivated by the general task of sampling motifs via the principle of maximum entropy. Ultimately, however, our approach allows us to derive *p*-value estimates and upper bounds which agree well with experiment over the entire domain of IC values without having to perform piece-wise approximations.

### False-positive identification in motif-finding settings

When we considered the problem of distinguishing true TF binding motifs from synthetic motifs of equivalent dimensions generated by Gibbs sampling, as in the left panel of Fig. 8, we found that the IGC percentile method can discriminate true positives at a rate consistently better than chance. This was true of synthetic false positives generated both from *E. coli* coding sequences and a random mononucleotide model, although the asymmetric nature of the ROC curve for synthetic motifs derived from *E. coli* suggests a depletion of low-IGC motifs in that negative set. We note that this may be an artifact of the use of coding sequences, where the degeneracy of the wobble position in-frame may increase the variance of conservation by column, relative to what would be expected by chance [72].

However, when we attempted to reproduce the performance of the ICG percentile classifier with synthetic motifs inferred by MEME (Fig. 8, right panel), we found that IGC percentile performed no better than chance at eliminating false positives. The reason for this result becomes clearer when we compare the distributions of IGC percentiles for synthetic motifs generated by MEME and Gibbs sampling in Fig. 9. While the IGC percentiles for Gibbs sampling motifs are roughly uniformly distributed, those for MEME motifs are strongly peaked about the maximum percentile value. This suggests a systematic bias in MEME towards motifs with higher IGC values, relative to what would be expected by chance, given IC.

This last finding should be interpreted as a caution, as it suggests that MEME may be less suited than methods based on Gibbs sampling to the discovery of low-IGC motifs. In the context of the TF binding site motif discovery problem which originally motivated the development of the MEME algorithm and provided its early test cases, such a bias is in fact generally helpful because TF binding motifs are also so biased (Fig. 4). In other motif-finding applications, however, the benefits of a bias towards high-IGC motifs is not as obvious, and might be taken into account when deliberating between MEME and Gibbs sampling methods which sample more evenly with respect to IGC.

### Limitations and future directions

#### Sample size

The runtimes of the algorithms presented in this work are cubic in the size of the motif, and this can present challenges for analyzing very large DNA motifs. In practice we have elected to downsample such large motifs to a regime where the algorithms are tractable, yet the small sample noise for the statistics of interest is negligible. For column-wise information content (and hence for IGC), this trade-off is not difficult to negotiate. For other choices of motif statistic, however, it is incumbent upon the user to ensure that the distribution of the statistic under downsampling is comparable to the statistic of the full motif.

#### Extension to the amino acid alphabet

In principle, the algorithms described above go through just as well for arbitrary alphabets other than nucleotides, including the standard amino acid alphabet. Although we have described the algorithms in this study in terms of the DNA alphabet for clarity, one may adapt them to any alphabet *A* rather mechanically, iterating over the elements of *A* where appropriate and replacing factors of 4 by factors of |*A*|.

We caution, however, that the time complexity is $\mathcal {O}(N^{|A|-1})$ in general, so that sampling for protein alignments in the standard amino acid alphabet goes as a 19^*th*^ degree polynomial. This may render analysis of large protein alignments quite taxing.

#### Extension to the continuous limit

The algorithms presented here are entirely discrete. When *N* is very large, however, it is tempting to pass to the continuous limit and treat the columns of a motif as elements of a probability simplex rather than as raw counts. MaxEnt distributions subject to mean IC over probability simplices are formally equivalent to the “entropic priors” of the form $P(\vec {p})\propto e^{-\beta H(\vec {p})}$ sometimes used in Bayesian inference [73]. However, to our knowledge there are no exact sampling algorithms for such distributions.

Another possibility is to construct a rejection sampling algorithm using a symmetric Dirichlet distribution as a proposal, since it is straightforward to sample [74] and the expected value of entropy can be found analytically [75]. Rejection sampling, however, requires the ratio of target and proposal densities to be globally bound by a known constant that, while empirically well-behaved, appears difficult to obtain in closed form.

## Conclusions

At root, bioinformatics is the study of patterns in biological sequence data. Such patterns appear largely as conserved elements in collections of sequences, with the degree of conservation quantified in terms of positional entropy. The description of such patterns has two essential aims, one basic and the other applied. In terms of basic research, one would often like to infer the existence of a biological mechanism from an unusual pattern in sequence data. In terms of applications, one often seeks to find more instances of a motif in a database of sequences, given a few positive examples. Searches based on information scores often suffer from large numbers of false positives, motivating the incorporation of additional features to further constrain a probabilistic model of the motif. Both of these tasks assume, however implicitly, that the practitioner can recognize an unusual feature of a collection of sequences, over and above what might be expected simply by chance from any collection with a similar degree of conservation. To our knowledge the statistical problem of sampling such collections has never been formally addressed.

In this study we provide algorithms for sampling from the maximum entropy distribution over nucleic acid motifs of a specified dimension given mean IC, as well as from the truncated uniform distribution over all motifs of a given dimension having IC within a given interval. Our methods allow researchers to ask, for any motif and motif statistic of interest, “how unusual is the observed value of the motif statistic among all similarly conserved motifs?” We note that in contrast to other strategies for sampling high-dimensional objects such as Markov Chain Monte Carlo methods, which generally provide samples from an approximation to the distribution of interest after long and typically uncertain runtimes, the algorithms described in this work are exact and efficient.

As one proof of concept, we investigated the distribution of IGC in a large and diverse collection of transcription factor binding motifs. The marked disparity of conservation between columns of binding motifs has long been noted, but until now there has been no principled way of quantifying the excess over what is due to chance. This would be especially difficult for IGC because it is a global statistic of a motif that does not reduce to lower-order functions of columns or column-pairs. Its null distribution is analytically intractable, and thus it illustrates the advantages of the parametric bootstrap framework. As another potential use-case, we show that the IGC percentile statistic can be used to help refine false positives from certain motif-finding algorithms. As a last illustration of the framework, we consider the motif *p*-value problem and show that it offers simple derivations of two powerful *p*-value estimators, as well as new interpretations of existing approaches.

## Additional file

Additional file 1 Contains a figure entitled “Motif Summary Statistics”, being a graphical representation of the data in Table 1. (208 KB PDF)

## Abbreviations

AUC: Area under curve
CDF: cumulative distribution function
erfcx: Complementary scaled error function
FDR: False discovery rate
FPR: False positive rate
GC: % Guanine + Cytosine content
IC: Positional information content
IGC: Informational Gini coefficient
MaxEnt: Maximum entropy distribution given information content
P.M.F.: Probability mass function
PSWM: Position-Specific Weight Matrix
ROC: Receiver operating characteristic
TPR: True positive rate
TU: Truncated uniform distribution given information content
