# Supplementary material for: Parametric bootstrapping for biological sequence motifs
Source: BMC Bioinformatics. 2016 Oct 6;17:406. doi: 10.1186/s12859-016-1246-8 (PMC5052923; doi:10.1186/s12859-016-1246-8)
Supplement: Additional file 1 — Contains a figure entitled “Motif Summary Statistics”, being a graphical representation of the data in Table 1. (208 KB PDF) [file 12859_2016_1246_MOESM1_ESM.pdf]

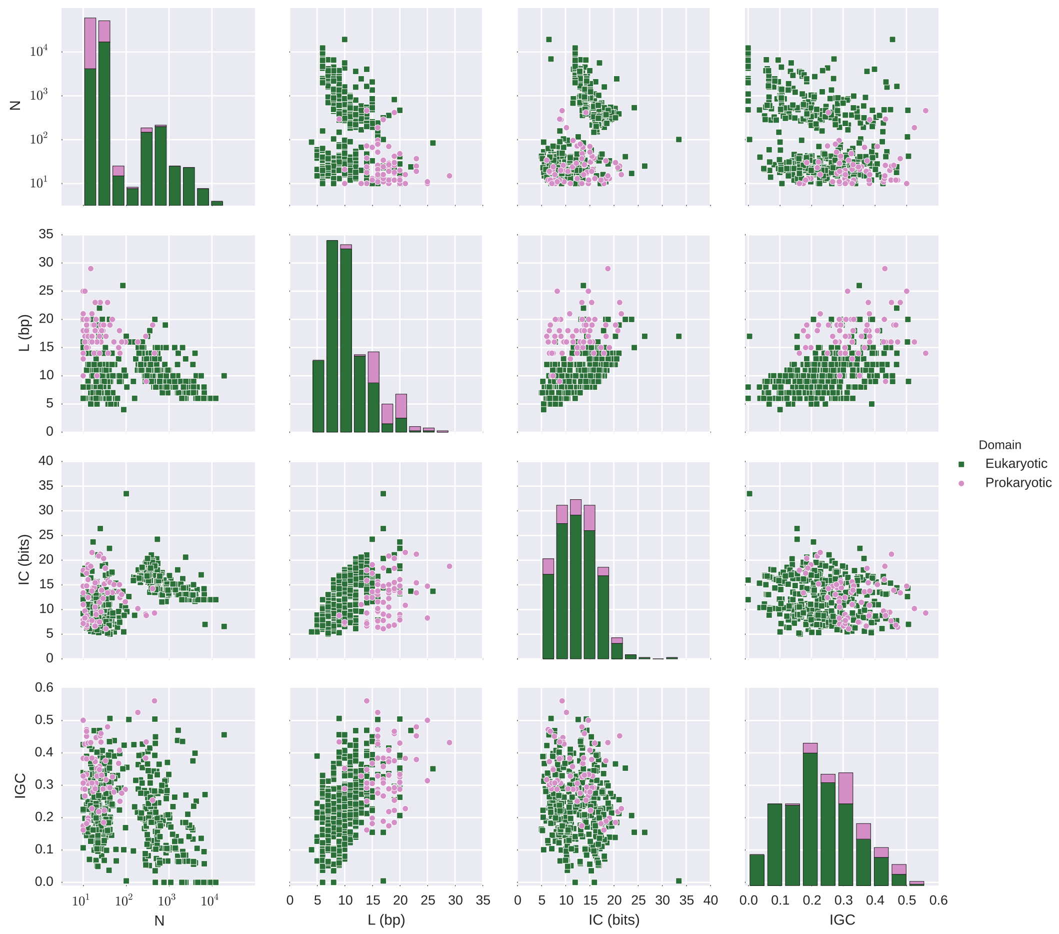

Motif Summary Statistics. Summary statistics from Table (1) are presented in graphical form. Diagonal elements depict histograms for each statistic, and off-diagonal elements depict scatterplots for each pair.
